# Mitochondrial dysfunction and insulin resistance: an update

**DOI:** 10.1530/EC-14-0092

**Published:** 2014-12-09

**Authors:** Magdalene K Montgomery, Nigel Turner

**Affiliations:** 1 Department of Pharmacology, UNSW Medicine, School of Medical Sciences, University of New South Wales, Kensington, Sydney, New South Wales, 2052, Australia

**Keywords:** mitochondrial function, insulin resistance, lipid accumulation, oxidative stress, mitophagy, mitochondrial dynamics

## Abstract

Mitochondrial dysfunction has been implicated in the development of insulin resistance (IR); however, a large variety of association and intervention studies as well as genetic manipulations in rodents have reported contrasting results. Indeed, even 39 years after the first publication describing a relationship between IR and diminished mitochondrial function, it is still unclear whether a direct relationship exists, and more importantly if changes in mitochondrial capacity are a cause or consequence of IR. This review will take a journey through the past and summarise the debate about the occurrence of mitochondrial dysfunction and its possible role in causing decreased insulin action in obesity and type 2 diabetes. Evidence is presented from studies in various human populations, as well as rodents with genetic manipulations of pathways known to affect mitochondrial function and insulin action. Finally, we have discussed whether mitochondria are a potential target for the treatment of IR.

## Mitochondrial function

**Figure 1:**
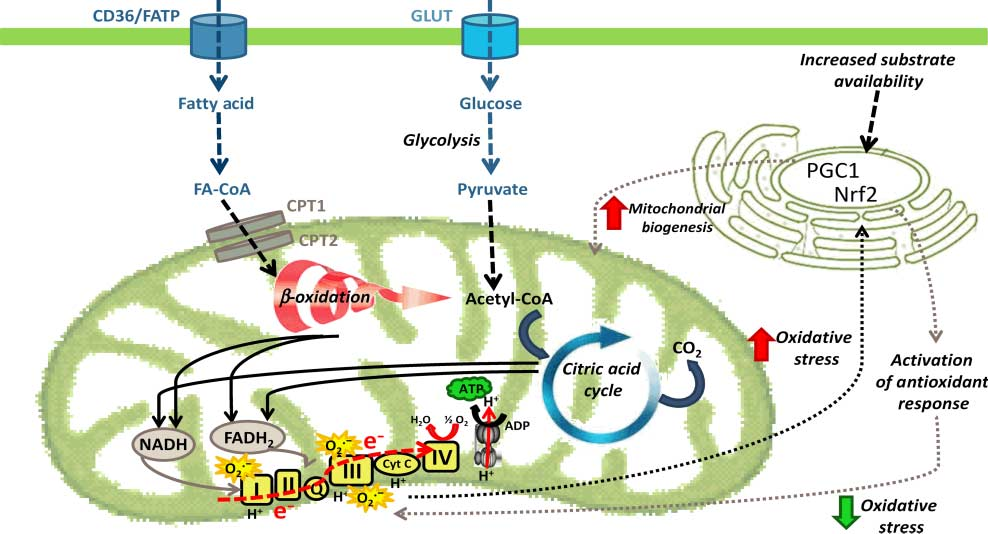
Fatty acids and glucose enter the cell via various membrane transporters. Fatty acids can either be converted to ‘active’ (DAG and ceramide) and ‘inert’ (TAG) lipid species or be transported into the mitochondria for oxidation to acetyl-CoA. Similarly, glucose can be metabolised to acetyl-CoA in mitochondria. Acetyl-CoA entering the citric acid cycle produces reducing equivalents (NADH and FADH_2_) that donate electrons for subsequent ATP generation in the electron transport chain. During electron transfer, superoxide (O_2_·^−^) is generated, causing oxidative stress and potential induction of *NRF2*, and activation of antioxidant response elements to decrease oxidative stress levels.

Mitochondria, originating from bacterial precursor cells that were able to generate energy, provide a platform for the generation of ATP, the energy currency of the cell. As ATP is essential for many cellular processes, mitochondrial function (and mitochondrial dysfunction) plays an important role in metabolic health and cellular fate. Mitochondrial function can be defined in a number of different ways, but for the purpose of this review we have largely focussed on the role of mitochondria in metabolic processes including oxidative phosphorylation and substrate oxidation (summarised in Fig. 1). The regulation of mitochondrial function is complex and still not fully understood. It involves rapid adaptations to changing metabolic conditions, such as fusion and fission, mitophagy as well as mitochondrial biogenesis. All of these will be discussed in the following sections, mainly in relation to changes in the obese state and under conditions of impaired insulin action (i.e. insulin resistance (IR)).

## Mitochondrial dysfunction

The diverse roles of mitochondria in different cellular processes and the multitude of methods used to examine mitochondrial function have led to variations in the definition of ‘mitochondrial dysfunction’. For example, mitochondrial function has been assessed by changes in mRNA levels of mitochondrial markers (either by targeted PCR or in more global microarray approaches) (1, 2, 3, 4), alterations in protein level (by immunoblotting) (1, 5) or in enzymatic activity of key components of mitochondria-driven oxidation (1, 6, 7, 8, 9), as well as changes in mitochondrial size and shape (by electron microscopy) (5, 7, 10) and substrate oxidation (6, 8). Accordingly, some groups referring to mitochondrial dysfunction as diminished mitochondrial content, others as a decrease in mitochondrial activity and oxidative phosphorylation, while others focus on different aspects such as reactive oxygen species (ROS) generation. In the context of this review, we refer to the term ‘mitochondrial dysfunction’ as a decrease in the mitochondrial oxidation of substrates, including lipid and carbohydrate, resulting from a general decrease in oxidative phosphorylation.

**Figure 2:**
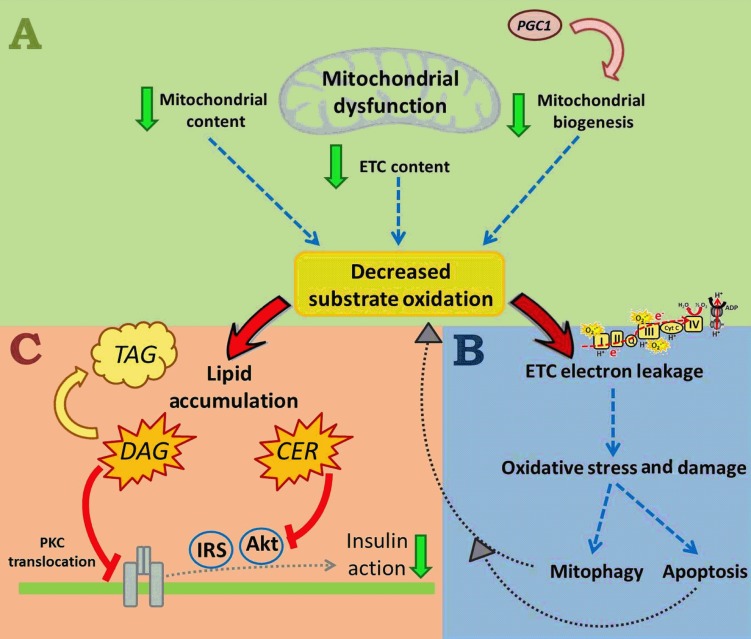
Mitochondrial dysfunction includes a reduction in mitochondrial content and mitochondrial biogenesis, and/or a decrease in the expression of mitochondrial oxidative proteins, such as complexes of the electron transport chain (ETC), with all those changes likely leading to decreased substrate oxidation (A). A diminished electron flow through the ETC can subsequently cause electron leakage and superoxide generation, followed by oxidative stress and damage. In a healthy environment, mitochondria can respond to damage through mitophagy pathways (removal of damaged mitochondria, preventing cell death), or in the case of high cellular stress, with apoptosis (B), both aggravating the decrease in substrate utilisation, and all up leading to increased lipid accumulation (C). Active lipid intermediates, such as diacylglycerols (DAG) and ceramide (CER) then cause inhibition of the insulin signalling pathway.

As well as defining mitochondrial dysfunction, it is important to give an overview about the possible mechanisms by which impairments in mitochondrial oxidative metabolism (i.e. mitochondrial dysfunction) could affect insulin sensitivity. Mitochondrial dysfunction can result from a decrease in mitochondrial biogenesis, reduced mitochondrial content and/or a decrease in the protein content and activity of oxidative proteins ‘per unit of mitochondria’ (such as a decrease in the complexes of the electron transport chain (ETC)) (Fig. 2). All such changes would presumably lead to a decrease in substrate oxidation (Fig. 2A). The reduced oxidation of fuels, particularly fatty acids, results in lipid accumulation, including deposition of metabolically active lipid mediators such as diacylglycerols (DAG) and ceramides (CER). Both DAG and CER have been shown to inhibit insulin signalling: DAG through protein kinase C activation translocates to the plasma membrane and inhibition of the insulin receptor (11), and CER through inhibition of the protein kinase AKT (Fig. 2C) (12, 13). DAG and CER accumulation is therefore a plausible link between mitochondrial dysfunction and IR. One potential caveat to this model is that muscle has an enormous spare respiratory capacity (i.e. oxygen consumption can increase ten to 20-fold above resting), and it has been proposed that a deficit in mitochondrial function of the magnitude observed in many obese and insulin-resistant individuals might not be expected to have major effects on substrate oxidation under resting conditions (14). However, it should be noted that in situations where muscle does dramatically increase respiration (e.g. exercise), there are typically large increases in muscle blood flow, marked changes in ATP demand, activation of multiple signalling pathways and large alterations in metabolite concentrations; in the absence of these changes (i.e. resting conditions), it is plausible that even relatively small decreases in substrate oxidation over time may partially lead to ectopic lipid accumulation and subsequently IR.

An additional potential mechanism linking mitochondrial dysfunction to IR is that decreases in substrate oxidation affect electron flow through the ETC, causing electron leakage towards oxygen and the formation of superoxide. Superoxide and other ROS damage various mitochondrial and cellular components (including oxidative damage to mitochondrial DNA, protein aggregations and lipid peroxidation), and potentially results in either mitophagy (removal of damaged mitochondria and prevention of cell death) or, under high stress levels, apoptosis (Fig. 2B). Removal of mitochondria through mitophagy could reduce mitochondrial number, resulting in decreased substrate oxidation, further aggravating lipid accumulation.

## The debate about mitochondrial dysfunction and IR

Mitochondrial dysfunction was first described in the context of glucose intolerance ∼40 years ago (15), and the majority of studies in this area since that time have focussed on changes in skeletal muscle, which will be the main organ discussed in this review. Several studies in humans (from the late 1990s) suggested the existence of mitochondrial dysfunction in obese and insulin-resistant patients, with these individuals exhibiting lower oxidative enzyme activities and decreased lipid metabolism in muscle compared with lean control subjects (6, 8, 9). In addition, Kelley *et al*. (7) published in 2002 that skeletal muscle of obese subjects with type 2 diabetes (T2D) exhibited lower NADH:O_2_ oxidoreductase activity and reduced mitochondrial size when compared with lean control subjects. One year later, two major microarray studies carried out in muscle showed mitochondrial biogenesis and oxidative phosphorylation pathways to be downregulated in T2D patients and non-diabetic individuals with a family history (FH+) of T2D when compared with healthy controls (2, 3). These two studies were of particular interest to metabolic researchers as i) they showed a decrease in peroxisome proliferator coactivator 1a (PGC1α), the master regulator of mitochondrial metabolism (Fig. 1), and therefore for the first time suggested a mechanism for the decrease in mitochondrial function, and ii) they provided evidence for genetic predisposition to mitochondrial defects and its occurrence in the ‘pre-diabetic’ state. Following these initial observations, several studies in humans showed similar downregulation of metabolic and mitochondrial pathways in obesity and IR (reviewed in (16 17)). Defects in the expression of mitochondrial genes were found at the mRNA level (1, 4, 10, 18) as well as at the protein level (1); this was accompanied by a decrease in oxidative enzyme activity (1, 5, 7) and mitochondrial size and density (5, 7, 10). To some extent, it still remains unclear whether the observed defects could be primarily due to a decrease in the number of mitochondria ‘per unit of muscle tissue’ or due to actual metabolic changes within the mitochondria (19, 20, 21, 22). Disparate results have also been reported with regards to the intramuscular populations of mitochondria that are affected (subsarcolemmal vs intermyofibrillar) (5, 23) and between different muscles across the body (24).

Although several human (as above) as well as rodent studies (25) have described associations between diminished mitochondrial function and obesity/IR, various independent publications have failed to show such a correlation. For example, several studies have shown that muscle mitochondrial function was not impaired in obese and T2D human subjects when compared with controls (19, 21, 26, 27). In addition, non-obese sedentary humans that were overfed for 28 days exhibited peripheral IR (determined as a decrease in glucose infusion rate during hyperinsulinemic–euglycaemic clamps) without changes in several markers of mitochondrial content in muscle (28). Similarly, rats fed a high-fat diet exhibited unchanged mRNA levels of various energy and glucose metabolism markers in muscle (29), as well as similar hepatic mitochondrial and peroxisomal fatty acid oxidation capacity when compared with low-fat diet controls (30).

Besides evidence for scenario 1 (a decrease in mitochondrial function with IR) and scenario 2 (unchanged mitochondrial function despite IR), several research groups, including ours, have shown a compensatory increase in mitochondrial oxidative capacity with increased lipid supply (31, 32, 33, 34). Mice and rats fed high-fat diets exhibited impairments in glucose tolerance and insulin sensitivity, but simultaneously an increase in fatty acid oxidative capacity, as well as protein content and activity of mitochondrial oxidative proteins in muscle (31, 32, 33, 34). Both the increase in mitochondrial content and oxidative capacity (35), as well as the development of IR (36), occur at around 3–4 weeks of high-fat feeding. Furthermore, in a recent comparison of mouse strain, our group showed that this mitochondrial adaptation to high-fat feeding was present in several different mouse strains (C57BL/6, 129X1, DBA/2 and FVB/N) that were prone to fat-induced obesity and glucose intolerance (37). The implications from these studies are that while there is a compensatory increase in mitochondrial oxidative capacity in rodents in response to dietary lipid oversupply, the timing and magnitude of these changes are not sufficient to cope with the dramatically enhanced lipid availability, and thus there is still ectopic lipid accumulation and IR. In support of this, dietary or genetic manipulations that enhance oxidative capacity in muscle above the normal adaptive response do ameliorate IR (32, 38, 39).

Collectively, the three possible scenarios described above (i.e. decreased, unchanged or a compensatory increase in mitochondrial function) suggest that mitochondrial dysfunction is not a requisite feature of IR in all circumstances and the presence of mitochondrial dysfunction is dependent on its definition, the population studied, the model system examined (e.g. human vs rodent models) and the methodological approach (e.g. association vs intervention studies).

## Alterations of mitochondrial function: effects on insulin sensitivity

As association studies of IR and mitochondrial function, as commonly reported for humans, are unable to delineate whether changes in mitochondrial function are a cause or consequence of IR, various groups have relied on genetic manipulations and interventional approaches to define the cause-or-consequence question of whether changes in mitochondrial function have effects on tissue and whole-body insulin sensitivity. Various transgenic approaches have been taken to try to answer this question. As it is not possible to cover all of these studies, we will give examples of alterations in gene expression of three key metabolic regulators, *TFAM*, *PGC1* and acetyl-CoA carboxylase 2 (*ACC2*), and discuss their effects on mitochondrial function and insulin sensitivity.

### TFAM

Mitochondrial biogenesis is a complex process, involving a coordinated regulation of mitochondrial and nuclear genomes. An important protein controlling the transcription of mitochondrial proteins is the nuclear-encoded transcription factor *TFAM*, whose expression is regulated by nuclear respirator factor 1 (Nrf1) (40). Manipulating the gene expression of this key regulator of mitochondrial transcription is therefore an excellent approach to alter mitochondrial function. Muscle-specific *Tfam*-knockout mice exhibit abnormally appearing mitochondria in their muscles as well as progressively deteriorating respiratory chain function (41). Interestingly, ATP levels are almost unchanged due to a substantial increase in mitochondrial mass (41). In a subsequent study, the same researchers investigated glucose homeostasis in muscle-specific *Tfam*-knockout mice and their results suggested that mitochondrial dysfunction in skeletal muscle is not a primary etiological event in the development of IR, as knockout mice exhibited improved glucose clearance during a glucose tolerance test and increased glucose uptake into muscle (likely due to increased expression of the glucose transporters GLUT1 and GLUT4 and increased AMP-activated protein kinase (AMPK) activity) (42). An independent group has recently created adipose tissue-specific *Tfam*-knockout mice, and their results are in line with the previous findings of Wredenberg *et al*. (43). As expected, *Tfam*-knockout mice exhibited decreased mtDNA copy number and diminished protein content of mitochondrially encoded proteins in brown and white adipose tissue, but displayed greater oxygen consumption due to higher uncoupling. Increased mitochondrial oxygen consumption resulted in elevated whole-body energy expenditure, which is a possible mechanism for these mice being protected against diet-induced obesity, glucose intolerance and lipid accumulation in their livers (43). Taken together, tissue-specific knockouts of *TFAM* indicate that experimentally inducing abnormalities in mitochondrial function does not necessarily lead to deterioration of glucose homeostasis and insulin sensitivity, highlighting a dissociation between IR and mitochondrial dysfunction.

### Peroxisome proliferator coactivator 1

Although the knockout of *TFAM* demonstrated a clear dissociation between mitochondrial function and insulin sensitivity, alterations in gene expression of members of the *PGC1* network have shown that metabolic downstream effects are variable, with complex compensatory interactions making interpretation of data difficult. *PGC1* *α* is a master-regulator of mitochondrial biogenesis, interacting with a large complement of transcription factors and nuclear hormone receptors that are associated with mitochondrial function (including *Nrf1*, *ERR* *α*, *YY1*, *PPAR* *α*, *PPAR* *γ*) (44). Global *Pgc1* *α*-knockout mice are viable; however they exhibit an abnormal energy metabolic phenotype. With age, *Pgc1* *α*-KO mice show an increase in whole-body as well as hepatic fat deposition, which is accompanied by a decrease in liver and muscle mitochondrial oxidative capacity. Surprisingly, KO mice were less susceptible to diet-induced IR than WT controls (45). Muscle-specific knockout of *PGC1* *α* also leads to increased fat mass; however in contrast, glucose tolerance was impaired in old mice (24 months), whereas it was unaltered in young (3 months) KO mice (46). This discrepancy between age groups may be explained by a compensatory increase in PGC1β in young, but not in aged mice (46). PGC1α and PGC1β regulate a large number of overlapping genes, therefore metabolic compensation is not surprising. In addition to *PGC1* *α* knockout, other studies have shown that also muscle-specific PGC1α overexpression causes IR and glucose intolerance, potentially due to increased fatty acid delivery into muscle and decreased *GLUT4* gene expression (47, 48). With these previous alterations of PGC1 protein levels, it should be noted that detrimental metabolic effects might be due to complicated adaptations due to life-long overexpression or knockout. In contrast, acute overexpression of *PGC1* *α* and *PGC1* *β* has shown that elevated PGC protein levels improve diet-induced IR in muscle (38, 49). Therefore, acute and targeted activation of PGC1-dependent pathways is likely to have therapeutic potential for the treatment of IR (50).

### Acetyl-CoA carboxylase 2

As lipid oversupply is a major factor involved in the development of IR, a prevailing view was that increasing mitochondrial fatty acid oxidation would improve insulin sensitivity. Mitochondrial fatty acid oxidation is, in part, regulated by AMPK, which phosphorylates and inactivates the mitochondrial enzyme ACC2, leading to reduced malonyl-CoA levels, elevated CPT1 activity and increased entry of long-chain fatty acids into mitochondria for oxidation (51). In a recent report by Hoehn *et al*. (51) activation of AMPK by the AMP-mimetic aminoimidazole carboxamide ribonucleotide (AICAR), as well as global *Acc2*-knockout in mice resulted in increased whole-body fatty acid oxidation. Interestingly, despite a significant increase in lipid oxidation, *Acc2*-knockout mice were not protected from high-fat diet-induced weight gain, glucose intolerance and deterioration of skeletal muscle glucose disposal, indicating that simply accelerating mitochondrial fatty acid oxidation alone does not prevent from a deterioration of insulin sensitivity (51). Similar observations were made by Olson *et al*. (52), who reported that selective deletion of *Acc2* in mouse skeletal muscle, as well as inactivation in the germline had no effects on body weight, food intake, body composition or glucose homeostasis as compared with controls on chow or high-fat diet. These recent studies of unchanged skeletal muscle and whole-body insulin sensitivity in *Acc2*-knockout mice contrast with earlier findings by Abu-Elheiga *et al*. (53), who reported that *ACC2* deletion leads to increased fat oxidation, reduced body weight and protection against diet-induced obesity and IR (54, 55, 56). Phenotypic differences observed in these different lines of *Acc2*-knockout mice could potentially be due to multiple experimental differences (e.g. genetic background, diet composition and cloning strategy), but highlight that seemingly identical genetic manipulations can give contrasting results, complicating advances in our understanding on the importance of mitochondrial function in the development of IR.

In the section above, we have noted a number of examples emphasising that due to variable adaptations in different mouse lines, more direct genetic manipulation of mitochondrial function has still been unable to clearly define the impact of altering mitochondrial capacity on tissue and whole-body insulin sensitivity. However, since many insulin-resistant individuals do show mitochondrial defects, it is possible that the mitochondrial defects could be a consequence of reduced insulin action, which is discussed below.

## Regulation of mitochondrial function by insulin

Insulin is an anabolic hormone that is known to play a major role in the regulation of metabolic pathways and protein synthesis in many tissues. A number of groups have examined the effect of insulin on mitochondrial function (largely via insulin infusions) and have also attempted to selectively alter glucose tolerance and insulin sensitivity in a previously ‘metabolically healthy’ environment to determine whether these changes affect mitochondrial function. The methodological approach taken for the latter has been to manipulate the components of the insulin signalling cascade to determine whether alterations directly affect insulin sensitivity (primary effect) and if changes in insulin sensitivity could potentially lead to alterations in mitochondrial capacity and function (secondary effect).

### Insulin infusions

Insulin is known to affect various metabolic pathways in lipid and glucose metabolism, by either affecting gene expression or the phosphorylation of metabolic proteins (57, 58, 59). In humans, several studies have also demonstrated direct effects of insulin to modify mitochondrial function, with insulin infusions leading to increased expression of mitochondrial proteins and higher oxidative enzyme activity and elevated ATP synthesis in muscle (60, 61). Further evidence of an effect of insulin on mitochondrial function came from a study by Karakelides *et al*. (62), who temporarily deprived type 1 diabetic patients of their insulin treatment and showed that insulin deficiency decreased muscle mitochondrial ATP production and expression of oxidative phosphorylation genes. This direct effect of insulin on mitochondrial function is diminished in subjects with IR (60) and in rats after high-fat feeding (63), suggesting that insulin is able to activate mitochondrial biogenesis and oxidative capacity, and that IR, particularly if it is present over a prolonged period, could in part contribute to mitochondrial dysfunction.

### Insulin receptor substrate gene manipulation

With regards to direct genetic manipulation of insulin signalling components, the best characterised models have manipulated insulin receptor substrate (IRS) proteins. In skeletal muscle, selective disruption of IRS1 or IRS2 had only minor effects on insulin sensitivity, whereas simultaneous deletion of both members of the insulin signalling cascade had dramatic effects on insulin signalling and glucose tolerance (64). Isolated muscles exhibited complete resistance to insulin and diminished insulin-stimulated glucose uptake. In addition, the lack of insulin signalling in double-KO muscle impaired mitochondrial oxidative phosphorylation and ATP production (64).

In addition to the findings in skeletal muscle, similar mitochondrial impairments were also observed in hepatocytes lacking both *IRS1* and *IRS2* (65). Cheng *et al*. (65) also found larger deformed and 50% fewer mitochondria in livers of these double-knockout (DKO) mice, with a simultaneous increase in markers of mitochondrial fission and fusion. Furthermore, many hepatic genes were deregulated in DKO livers, including genes known to control mitochondrial function, biogenesis and dynamics (65). Also, similar to the metabolic changes in skeletal muscle, selective silencing of *IRS1* or *IRS2* in liver had only minor effects on insulin signalling, whereas disruption of both isoforms led to impaired glucose tolerance and insulin sensitivity (66).

These results in muscle and liver suggest that direct genetic induction of IR has major effects on mitochondrial capacity, and that IR itself is able to lead to the development of mitochondrial dysfunction. Mitochondrial effects of *IRS1/2* deletion are likely mediated through the IRS-PI3K-Foxo pathway, as additional deletion of *Foxo1* (triple knockout) was able to rescue the metabolic phenotype (65).

These studies of genetic manipulation of components of the insulin-signaling cascade, as well as direct insulin infusion or insulin deprivation, raise the possibility that mitochondrial defects observed in some insulin-resistant subjects could be a consequence of the IR itself.

## Ectopic lipid accumulation: effects on other mitochondrial parameters

Ectopic lipid accumulation is commonly observed in obesity and T2D and is one of the earliest changes observed during the development of IR (36). Mitochondria respond to cellular stress (including excess lipid availability) in various ways, for example through increase in reactive species generation, complementation by fusion and fission and activation of cell death pathways. Interestingly, cells have developed a mechanism to sequester and degrade non-functional mitochondria, a process termed mitophagy, before cell death can occur. In this section, we will discuss scientific findings with regards to excess lipid availability and IR and the role of mitochondrial oxidative stress, mitophagy and stress mitigation through mitochondrial fusion and fission pathways.

### Oxidative stress

Mitochondria are an important source of superoxide generation in the cell, with complexes I and III of the ETC having the greatest capacity for superoxide production (67). Most ROS within cells originate from superoxide and hydrogen peroxide, a product resulting from degradation of superoxide by the superoxide dismutase enzymes, or from biochemical reactions with these primary ROS (68). Under physiologically relevant ADP-stimulated conditions, mitochondrial superoxide represents only around 0.018% of total oxygen consumption (69), being therefore ‘concentration-wise’ a minor but ‘implication-wise’ a major player in mitochondrial function.

Several studies from the 1990s proposed a link between oxidative stress and the development of IR. Lipid infusions in humans increased plasma thiobarbituric acid reactive substance (TBARS) levels and simultaneously inhibited insulin-stimulated whole-body glucose disposal (70). In contrast, infusions of reduced glutathione exhibited the opposite effect on both oxidative damage and insulin sensitivity (70, 71).

More direct evidence connecting IR and mitochondrial ROS generation came from a recent study by Anderson *et al*. (72). In both rodents and humans, a high-fat diet increased the H_2_O_2_-emitting potential of mitochondria without any changes in oxidative capacity. Attenuating mitochondrial H_2_O_2_ emission, by treating rats with a mitochondrial-targeted antioxidant or via overexpression of catalase in mouse muscle mitochondria, completely preserved glucose tolerance and insulin sensitivity (72). Similar beneficial effects for insulin action were observed when mitochondrial superoxide was reduced by genetic or pharmacological means (73). These and various other *in vitro* studies (74, 75) and genetic manipulations of antioxidant expression (e.g. targeted overexpression of catalase to mitochondria (76) or overexpression of mitochondrial peroxiredoxin 3 (77) highlight mitochondrial superoxide and H_2_O_2_ as a primary mechanism in the development of IR.

The studies noted above support a role for oxidative stress in the development of IR, and there have been a number of antioxidant supplementation trials which have reported that decreasing oxidative stress improves insulin sensitivity and glucose tolerance in various human populations (78, 79, 80, 81, 82) and animal models (83, 84, 85, 86). However, it should be noted that there is still substantial controversy in this area, as various independent studies have failed to show such association. For example, antioxidant supplementation had minimal impact on insulin sensitivity in various human populations (87, 88, 89, 90). Similarly, directed reduction in mitochondrial ROS generation using a mitochondria-targeted antioxidant did reduce oxidative stress levels in C2C12 myotubes as well as in mice, but did not affect glucose tolerance and insulin sensitivity (91).

These disparate findings suggest that depending on the experimental conditions, oxidative stress may be one of a number of factors contributing to IR. However, in circumstances where elevated mitochondrial ROS production is present during the development of IR, it is likely tightly coupled with autophagic removal of damaged mitochondria, a process termed mitophagy.

### Mitophagy

Upon increased cellular and mitochondrial stress, mitochondria are able to activate both cell death pathways and mitophagy, with the two opposing forces in the cell, demonstrating a tight balance between life and death (92). The targeted removal of damaged mitochondria requires two steps: the initiation of autophagy and selective priming of mitochondria for removal (93). Priming is carried out by either the Pink1–Parkin pathway or through the activation of Nix and Bnip3 (94). Under metabolically healthy conditions, Pink1 is rapidly cleaved by mitochondrial proteases and degraded by the proteasome. However, upon loss of mitochondrial membrane potential Pink1 accumulates on the outer mitochondrial membrane and communicates with the E3 ubiquitin ligase Parkin. Parkin rapidly translocates to mitochondria and signals the initiation of mitophagy by ubiquinating mitochondrial proteins (VDAC1, mitofusin 1, mitofusin 2 and MIRO) (93). Furthermore, Parkin recruitment is suggested to be dependent on translocation of HSP72 to depolarised mitochondria, with HSP72-knockout mice exhibiting impaired Parkin action, enlarged and dysmorphic mitochondria, reduced muscle oxidative capacity, and muscle IR (95). This is one of few studies potentially suggesting mitophagy to be a beneficial metabolic event, important for the maintenance of mitochondrial quality and insulin sensitivity.

### Mitochondrial fusion and fission

To maintain a metabolically healthy environment, removal of damaged mitochondria through mitophagy requires cells to be able to adapt quickly through changes in mitochondrial fusion and fission pathways. Mitochondria are dynamic organelles able to constantly undergo fusion and fission events to maintain their function (96). Fusion of mitochondria enables them to mix their contents, including mitochondrial DNA and metabolic intermediates, and to recover the activity of damaged or depolarised membranes (97). Mitochondrial fusion is predominantly controlled by three GTPases, mitofusin 1 and 2* (Mfn1*/2), both located on the outer mitochondrial membrane, and optic atrophy1 (*Opa1*), localised on the inner membrane of mitochondria (98, 99). Fission on the other hand increases the number of mitochondria and prepares the cell for cell division and meiosis (100). It is regulated through the activity of *Fis1*, located throughout the outer membrane, and the dynamin-related protein 1 (DRP1). DRP1, lacking a mitochondrial targeting sequence, is usually located in the cytosol, but gets recruited to the mitochondrial outer membrane by *Fis1* during the initiation of the fission process (101). Changes in mitochondrial dynamics could contribute to mitochondrial dysfunction observed in certain obese and insulin-resistant human and rodent populations. Skeletal muscle of obese and T2D humans and rodents shows reduced *MFN2* expression (reviewed in (102)). Silencing of *MFN2* in myotubes leads to decreased oxygen consumption and glucose oxidation (103, 104), with a simultaneous decrease in glucose incorporation into glycogen, as well as reductions in pyruvate and palmitate oxidation in myotubes (104). Changes in oxygen consumption and substrate oxidation with reduced *MFN2* are likely due to a decrease in the expression of subunits of the electron transport complexes, which leads to reduced activity (102). These metabolic effects of *MFN2* are independent of its role in mitochondrial fusion, as a mutant form of mitofusin 2, lacking the ability to induce fusion, was still able to stimulate energy metabolism (104). Decreased oxygen consumption and mitochondrial dysfunction were also observed *in vivo* in mice with simultaneous deletion of *Mfn1* and *Mfn2*, which was suggested to be related with mtDNA depletion in early stages of life before physiological abnormalities developed (105). Interestingly, separate deletion of *Mfn1* or *Mfn2* did not result in any metabolic abnormalities (e.g. normal cytochrome c oxidase/succinate dehydrogenase (COX/SDH) histological staining pattern and no indication of respiratory deficiency), suggesting possible compensatory interactions between both isoforms (105). Due to the observed role of mitofusins in metabolism and their reported decrease in obesity and IR, these major regulators of mitochondrial dynamics are potentially important mediators of mitochondrial dysfunction. This is also supported by the fact that *Mfn2* levels show a positive correlation with insulin sensitivity in morbidly obese subjects and after bariatric surgery (106, 107).

## Targeting mitochondria to treat obesity and IR

As described in the preceding sections, there is substantial controversy on the exact role of mitochondria in the generation of IR. However, because of their role as the key site for substrate oxidation and ATP generation, targeting mitochondrial energy metabolism may has some benefit for metabolic disorders such as obesity and T2D. Below we discuss the evidence for various approaches (lifestyle interventions that affect mitochondrial oxidative metabolism and insulin action and more direct alterations of mitochondrial oxidative metabolism) aimed at improving aspects of mitochondrial function for potential therapeutic purposes. We also highlight the intriguing work showing that under certain circumstances, mild inhibition of mitochondrial function can also result in beneficial metabolic effects.

### Lifestyle interventions

Exercise is known to have major impact on both mitochondrial function and insulin sensitivity in skeletal muscle. A 12-week exercise intervention programme in T2D patients significantly increased muscle mitochondrial respiration and mitochondrial content (108). Similarly, an 8-week cycling exercise regime increased muscle fatty acid oxidative capacity and in parallel improved insulin-mediated glucose disposal (109). An improvement in insulin sensitivity and mitochondrial function (mitochondrial density and oxidative enzyme activity) was also reported by Toledo *et al*. (110) in T2D patients after 4 months of exercise training, by Nielson *et al*. (111) in male T2D subjects after 10 weeks of exercise, and by Meex *et al*. (112) in T2D patients after a 12-week exercise bout.

In addition to exercise, caloric restriction is an effective treatment improving obesity and IR. In obese humans, caloric restriction and weight loss were shown to improve insulin action (113, 114, 115), reaching a 40% improvement in insulin sensitivity after 6 months of caloric restriction (116). Calorie restriction has also been reported to enhance mitochondrial function in humans (117). The NAD^+^-dependent deacetylase sirtuin 1 (SIRT1) is suggested to be the principal modulator of the downstream pathways responsible for the beneficial metabolic effects of calorie restriction (118, 119). In line with this, the SIRT1 activator resveratrol increases mitochondrial content, ameliorates IR and prolongs survival (120, 121). Recently, novel compounds have been characterised, which are 1000-fold more potent than resveratrol (122), with these compounds inducing a robust increase in mitochondrial content and respiration, which is associated with improved insulin sensitivity in mice (123, 124).

### Mitochondrial uncoupling

The coupling of mitochondria describes the molar ratio of the yield of ATP (per oxygen consumed), also referred to as the P/O ratio, while mitochondrial uncoupling is defined as futile cycling of protons across the mitochondrial inner membrane, without coupling to ATP generation (125). The inefficiency induced by mitochondrial uncoupling leads to increased energy expenditure and mitochondrial substrate oxidation, as well as improved insulin action (126, 127, 128).

Pharmacological agents, such as 2,4-dintirophenol (DNP), can induce mitochondrial uncoupling by transporting protons across the mitochondrial inner membrane into the matrix. DNP was successfully used as an anti-obesity agent in the 1930s, suggesting that increased energy expenditure through mitochondrial uncoupling could have therapeutic potential (129). However, due to its narrow therapeutic window, overdoses led to serious imbalances of energy metabolism (and even to death), and DNP had to be taken off the market in 1938. More recently described uncoupling agents may potentially have a safer profile for use in humans (130, 131, 132). For example, derivatives of rhodamine 19 were shown to reduce membrane potential and increase oxygen consumption. However, In contrast to DNP, the activity of those derivatives was highest at high membrane potentials and decreased with a loss of membrane potential, therefore exhibiting self-limitation and potentially being a safer option than DNP (130). In addition, Lou *et al*. (131) identified various mitochondrial uncouplers, that appear to act through the adenine nucleotide translocase and have a much greater therapeutic window than previously observed with DNP (dynamic range of 10(6)).

### Natural compounds

Asian countries have a long history of using natural compounds for the treatment of metabolic disease. One of the compounds is Berberine, a natural plant alkaloid that was shown to improve insulin sensitivity in rodents and humans (133, 134). Interestingly, the effects of Berberine are not through enhancement of mitochondrial function (as attempted with many other therapies), but appear to be through inhibition of complex I of the ETC, subsequent AMPK activation and the consequent beneficial metabolic effects (135). Interestingly, this counter-intuitive pattern of mild inhibition of mitochondrial oxidative metabolism has been reported in other insulin-sensitising medicinal plants (136) and is also a characteristic of metformin and thiazolidinediones, which are frontline anti-diabetic therapies (135, 137, 138). While it is not completely resolved how mild, transient inhibition of mitochondrial function can lead to beneficial effects, it likely involves a number of mechanisms including reductions in deleterious lipid metabolites (due to decreased lipid synthesis and increased fatty acid oxidation following AMPK activation) and potentially decreases in ROS production (139). Increased fatty acid oxidation, despite a mild inhibition of complex I, is likely to occur due to AMPK-mediated lowering of malonyl-CoA levels and increased CPT1-mediated fatty acid entry into mitochondria. Increased flux of fatty acids into β oxidation generates both NADH and FADH_2_, with the entry of electrons into the ETC via complex II allowing substrate metabolism to continue despite a transient block at complex I. In addition, a decrease in ROS generation could be potentially mediated via reduced electron backflow and superoxide production from the ubiquinone reduction site of complex I with FADH_2_ (generated during each β oxidation cycle) as the electron donor.

## Concluding remarks

During the last 20 years, many studies have reported changes in mitochondrial function in various models of metabolic disease. Interestingly, these changes have been of opposing character, with some studies reporting a decrease in mitochondrial function (i.e. mitochondrial dysfunction), while others have reported either no change or an increase in mitochondrial oxidative metabolism in obese and insulin resistant humans and rodents. Similarly, studies using genetically manipulated mice to alter mitochondrial function have also produced confounding results. Collectively, these studies highlight that the relationship between mitochondria and insulin action is highly complex and there is still much to learn in this area. Some potential approaches for closing these gaps in knowledge in future studies include developing methods (e.g. with stable isotope tracers) to assess dynamic changes in mitochondrial function *in vivo*, not just in the typical fasting state, but also under a variety of other free-living conditions (e.g. in response to meals and during normal daily activities). Furthermore, increasing evidence of multiple post-translational modifications in mitochondria highlights that future studies in isolated mitochondria or tissue preparations must be performed in the presence of appropriate inhibitors to maintain the mitochondria in a similar state that observed *in vivo*. Studies such as these may help to more clearly define the extent to which mitochondrial dysfunction contributes to the development of IR, and also determine how effective targeting mitochondria may be for the treatment of IR.
